# Recent Research

**Published:** 1890-09-20

**Authors:** 


### RECENT RESEARCH.

Dr. John William Hunter, Research student of the University of Cambridge, whose views on pernicious anæmia we referred to in the Retrospect of February 12th, has now given his observations on the excretion of ptomaines in pernicious anæmia. He details in the *British Medical Journal* the method he employed to determine if cadaveric poisons or ptomaines are passed in the urine of this disease. He succeeded in isolating a quantity of a double benzoyl compound. As to the significance to be attached to the presence of ptomaines in the urine, Dr. Hunter speaks with reserve. He is inclined, however, to attach not a little importance to it, for such things are not found in the urine in health. As they are only found by the action of the micro-organisms, he concludes that their presence in the urine points to the action of special micro-organisms, and Dr. Hunter asks, what is the relation between the excretion of these bodies in the urine and symptoms observed in pernicious anæmia. He says the exacerbations from which his patient suffered presented the phenomena of toxic poisoning, regard being had to nervous phenomena—the sudden onset of drowsiness, contracted pupils, slight fever, and sweating—or to the more local manifestations of the action of such poisons, the intestinal disturbance, and the obviously-increased destruction of blood which always attended such attacks. Dr. Hunter thinks that the alimentary canal is the seat of the production of toxic agents in pernicious anæmia. He is disposed to regard the gastric mucosa as the seat, not only of primary development of specific micro-organisms—a development which may be favoured by an unhealthy condition of the stomach and the tongue. Thus, Dr. Hunter comes to the conclusion that the special factor required, to initiate the symptoms peculiar to the special disease, pernicious anæmia, is the presence under favourable conditions of organisms of specific nature within the gastro-intestinal tract. Dr. Hunter's paper is a model of close reasoning, but as the conclusions are based on the history of one case, we await further development and research.

